# Simulation-guided pan-cancer analysis identifies a novel regulator of CpG island hypermethylation heterogeneity

**DOI:** 10.1093/bib/bbaf252

**Published:** 2025-06-02

**Authors:** Xianglin Zhang, Wei Zhang, Jinyi Zhang, Xiuhong Lyu, Haoran Pan, Tianwei Jia, Ting Wang, Xiaowo Wang, Haiyang Guo

**Affiliations:** Department of Clinical Laboratory, the Second Hospital, Cheeloo College of Medicine, Shandong University, 247 Beiyuan Street, Tianqiao District, Jinan, 250033, Shandong, China; Shandong Engineering & Technology Research Center for Tumor Marker Detection, Department of Clinical Laboratory, the Second Hospital, Cheeloo College of Medicine, Shandong University, 247 Beiyuan Street, Tianqiao District, Jinan, 250033, Shandong, China; Shandong Provincial Clinical Medicine Research Center for Clinical Laboratory, Department of Clinical Laboratory, the Second Hospital, Cheeloo College of Medicine, Shandong University, 247 Beiyuan Street, Tianqiao District, Jinan, 250033, Shandong, China; Center of Intelligent Medicine, School of Control Science and Engineering, Shandong University, 17923 Jingshi Road, Lixia District, Jinan, 250061, Shandong, China; Department of Clinical Laboratory, the Second Hospital, Cheeloo College of Medicine, Shandong University, 247 Beiyuan Street, Tianqiao District, Jinan, 250033, Shandong, China; Shandong Engineering & Technology Research Center for Tumor Marker Detection, Department of Clinical Laboratory, the Second Hospital, Cheeloo College of Medicine, Shandong University, 247 Beiyuan Street, Tianqiao District, Jinan, 250033, Shandong, China; Shandong Provincial Clinical Medicine Research Center for Clinical Laboratory, Department of Clinical Laboratory, the Second Hospital, Cheeloo College of Medicine, Shandong University, 247 Beiyuan Street, Tianqiao District, Jinan, 250033, Shandong, China; Department of Emergency Medical Center, the Second Hospital, Cheeloo College of Medicine, Shandong University, 247 Beiyuan Street, Tianqiao District, Jinan, 250033, Shandong, China; Department of Clinical Laboratory, the Second Hospital, Cheeloo College of Medicine, Shandong University, 247 Beiyuan Street, Tianqiao District, Jinan, 250033, Shandong, China; Shandong Engineering & Technology Research Center for Tumor Marker Detection, Department of Clinical Laboratory, the Second Hospital, Cheeloo College of Medicine, Shandong University, 247 Beiyuan Street, Tianqiao District, Jinan, 250033, Shandong, China; Shandong Provincial Clinical Medicine Research Center for Clinical Laboratory, Department of Clinical Laboratory, the Second Hospital, Cheeloo College of Medicine, Shandong University, 247 Beiyuan Street, Tianqiao District, Jinan, 250033, Shandong, China; Department of Clinical Laboratory, the Second Hospital, Cheeloo College of Medicine, Shandong University, 247 Beiyuan Street, Tianqiao District, Jinan, 250033, Shandong, China; Shandong Engineering & Technology Research Center for Tumor Marker Detection, Department of Clinical Laboratory, the Second Hospital, Cheeloo College of Medicine, Shandong University, 247 Beiyuan Street, Tianqiao District, Jinan, 250033, Shandong, China; Shandong Provincial Clinical Medicine Research Center for Clinical Laboratory, Department of Clinical Laboratory, the Second Hospital, Cheeloo College of Medicine, Shandong University, 247 Beiyuan Street, Tianqiao District, Jinan, 250033, Shandong, China; Department of Genetics, The Edison Family Center for Genome Sciences and Systems Biology, Washington University School of Medicine, 4515 McKinley Avenue, St. Louis, MO 63110, United States; McDonnell Genome Institute, Washington University School of Medicine, 4444 Forest Park Avenue, St. Louis, MO 63108, United States; Ministry of Education Key Laboratory of Bioinformatics; Center for Synthetic and Systems Biology; Bioinformatics Division, Beijing National Research Center for Information Science and Technology; Department of Automation, Tsinghua University, No 1 Qinghuayuan Street, Haidian District, Beijing 100084, China; Department of Clinical Laboratory, the Second Hospital, Cheeloo College of Medicine, Shandong University, 247 Beiyuan Street, Tianqiao District, Jinan, 250033, Shandong, China; Shandong Engineering & Technology Research Center for Tumor Marker Detection, Department of Clinical Laboratory, the Second Hospital, Cheeloo College of Medicine, Shandong University, 247 Beiyuan Street, Tianqiao District, Jinan, 250033, Shandong, China; Shandong Provincial Clinical Medicine Research Center for Clinical Laboratory, Department of Clinical Laboratory, the Second Hospital, Cheeloo College of Medicine, Shandong University, 247 Beiyuan Street, Tianqiao District, Jinan, 250033, Shandong, China

**Keywords:** DNA methylation, heterogeneity, CpG island methylator phenotype, tumor purity, negative association identification

## Abstract

CpG island hypermethylation, a hallmark of cancer, exhibits substantial heterogeneity across tumors, presenting both opportunities and challenges for cancer diagnostics and therapeutics. While this heterogeneity offers potential for patient stratification to predict clinical outcomes and personalize treatments, it complicates the development of robust biomarkers for early detection. Understanding the mechanisms driving this heterogeneity is essential for advancing biomarker design. Here, simulation-based analyses demonstrate that tumor purity and the high prevalence of low epi-mutation samples significantly obscure the identification of negative, rather than positive, regulators of CpG island hypermethylation, limiting a comprehensive understanding of heterogeneity sources. By addressing these confounders, we identify impaired DNA methylation maintenance, as indicated by global hypomethylation levels, as the primary contributor to CpG island hypermethylation variability among known regulators. This finding is supported by integrative analyses of datasets from The Cancer Genome Atlas (TCGA) Pan-Cancer Atlas, Genomics of Drug Sensitivity in Cancer (GDSC1000) cancer cell lines, and epi-allele analyses of two independent whole-genome bisulfite sequencing cohorts, using a newly developed method, MeHist (https://github.com/vhang072/MeHist). Furthermore, we assess widely used hypermethylation biomarkers across ten cancer types and find that 65 out of 246 (26.4%) are significantly influenced by impaired methylation maintenance. Incorporating hypomethylation and hypermethylation markers improves the robustness of cancer detection, as validated across multiple plasma cell–free DNA datasets. In summary, our findings highlight the value of simulation-guided integrative analysis in mitigating confounding effects and identify impaired DNA methylation maintenance as a key regulator of CpG island hypermethylation heterogeneity.

## Introduction

CpG islands (CGIs) are genomic regions spanning hundreds to thousands of bases, enriched for CpG dinucleotides, and serving as key *cis*-regulatory elements for housekeeping and developmental genes in mammalian genomes [1, 2]. The epigenetic states of CGIs play critical roles in regulating gene transcription and determining cell fate. In cancer cells, CGIs often exhibit hypermethylation, which silences promoter-associated genes, thereby influencing cancer cell viability and reshaping the tumor microenvironment [3–6]. For example, hypermethylation of the 5′ CGI in the DNA mismatch repair gene *MLH1* leads to microsatellite instability in colorectal and endometrial cancers [7, 8].

A hallmark of CGI hypermethylation is its high heterogeneity across tumors. The CpG island methylator phenotype (CIMP) has been proposed to represent a subset of tumors with widespread CGI hypermethylation [3, 9–14]. While this heterogeneity holds promise for patient stratification, enabling prediction of clinical outcomes and development of tailored therapies [5, 6, 15, 16], it complicates the design of robust biomarkers for early cancer detection [17, 18]. Understanding the mechanisms underlying CGI hypermethylation heterogeneity is thus essential for advancing biomarker development.

In the human genome, the DNA methylation landscape is regulated by three key processes: active *de novo* methylation mediated by DNMT3A, DNMT3B, and DNMT3L (a cofactor without enzymatic activity), active demethylation by TET enzymes (TET1, TET2, TET3), and DNA methylation maintenance during replication by DNMT1 and its cofactor UHRF1 [19–22]. Perturbations in these processes can theoretically disrupt methylation patterns and contribute to the observed heterogeneity in cancer cells. Prior studies have identified several factors influencing CGI hypermethylation heterogeneity, including alterations in genes regulating methylation (e.g. *DNMT3A*, *IDH1*, *IDH2*, *TET*) [9, 23–28], viral infections [3], cell proliferation rates [28–30], chronic inflammation [31], and reduced TET activity under tumor hypoxia conditions [32]. However, these factors are often cancer type specific and collectively explain only a fraction of the observed heterogeneity (~14%) [32], suggesting the presence of unidentified regulatory mechanisms.

Genome-wide hypomethylation, particularly in CpG-depleted regions such as retrotransposons and solo-WCGW sites, represents another hallmark of cancer and is attributed to impaired methylation maintenance during cell division [33, 34]. While clinical studies suggest that CGI hypermethylation and genome-wide hypomethylation are independent processes [12, 35–37], experiments in cancer cell lines have shown that extreme methylation maintenance loss (e.g. via disruption of DNMT1 or UHRF1) can reverse CGI hypermethylation [19, 21]. This apparent paradox raises the question of whether variations in methylation maintenance across tumors contribute to CGI hypermethylation heterogeneity and, if so, to what extent.

In this study, we address this question through simulations and comprehensive analyses of large-scale datasets. We account for confounding factors such as tumor purity and the prevalence of tumors with low epi-mutation burdens, confirming that deficiencies in methylation maintenance significantly attenuate CGI hypermethylation and explain the largest proportion of its variability among known regulators. Notably, we observed that high epi-mutation burden correlates with poor clinical outcomes across 10 cancer types. Furthermore, we evaluated well-established hypermethylation biomarkers for liquid biopsy and found that 26.4% of these markers are significantly attenuated in tumors with low methylation maintenance, underscoring the importance of incorporating hypomethylation and hypermethylation markers in cancer detection. This finding was validated using multiple plasma cell–free DNA datasets.

## Materials and methods

### DNA methylation analysis

We calculated the average values of normal counterparts for each cancer type and defined probes in tumor samples that deviate by ≥0.2 from these normal means as potential differential probes [10]. Using this method, we determined the number of differential probes for each tumor sample. Hypermethylated and hypomethylated probes were further filtered through two steps: (a) the normal mean values of hypermethylated probes must be <0.3, while those of hypomethylated probes must exceed 0.7; (b) the fraction of hypermethylated tumors for each probe must be >5% for TCGA data and 10% for cancer cell line data to exclude the potential influence of atypical samples. The normal samples of PCPG, KIRC, and GBM were used as normal counterparts for ACC, KICH, and LGG, respectively, due to their organ-of-origin similarity.

Whole-genome bisulfite sequencing (WGBS) data were mapped to the human reference genome GRCh38 using Bismark [38]. Reads mapped to CGIs were extracted and analyzed at epi-allele level. The distributions of mean methylation of bisulfite sequencing reads were calculated for each CpG island, and RIN-hyper was defined as the ratio of incompletely methylated reads (0.5 < methylation < 0.9) to completely methylated reads (methylation > 0.9). RIN-hyper can reflect the pattern and the heterogeneity of hypermethylated CGIs. The implemented method has been deposited on GitHub (https://github.com/vhang072/MeHist).

To assess the specific advantages of hypomethylation and hypermethylation biomarkers for cancer detection, we analyzed plasma cell–free DNA datasets generated using either WGBS or targeted/enriched bisulfite sequencing. Due to the low coverage and instability of individual biomarkers, we computed the average signal across all hypermethylation and hypomethylation biomarkers, respectively, to evaluate their specific advantages in distinguishing cancer patients from non-cancer individuals.

### Gene expression analysis

This study utilized three gene signatures: “Hypoxia” (*ALDOA*, *MIF*, *TUBB6*, *P4HA1*, *SLC2A1*, *PGAM1*, *ENO1*, *LDHA*, *CDKN3*, *TPI1*, *NDRG1*, *VEGFA*, *ACOT7*, and *ADM*), “Proliferation” (*MKI67*, *NDC80*, *NUF2*, *PTTG1*, *RRM2*, *BIRC5*, *CCNB1*, *CEP55*, *UBE2C*, *CDC20*, and *TYMS*), and “Cell Cycle” (gene list from reactome Cell Cycle R-HSA-1640170, 671 genes, https://reactome.org/content/detail/R-HSA-1640170) [32, 39]. Notably, 9 out of 11 genes in the “Proliferation” signature are included in the “Cell Cycle” signature, with the exceptions of *MKI67* and *CEP55*. Gene expression values were transformed into *Z*-scores, and the mean *Z*-score for each gene list was used as the final gene signature output for each sample. Gene enrichment analysis was conducted using Metascape [40].

### Simulation

Simulations were conducted in quadrant I, meaning the alterations of the two variables were transformed into positive values. We set 100 simulation points, comparable to the number of samples from a single cancer type in TCGA. For the positive correlation, changes in variable 1 (ΔV1) were randomly generated following a uniform distribution. Changes in variable 2 (ΔV2) were generated to follow a positive correlation (*R* = 1) with ΔV1, incorporating random residues within ±10% of the ΔV1 range. These random residues simulated variations that were independent of variable 1. Tumor purity was generated following a uniform distribution ranging from 0.1 to 1 and was multiplied by the generated ΔV1 and ΔV2.

For the negative correlation, similar operations were conducted, establishing a negative correlation (*R* = −1) between the two variables (pattern N1). Two additional admixture simulations were conducted to mimic conditions where two subtypes of tumor samples (low and high epi-mutation burden) coexist within the same cancer types. Two sets of points were simulated: one following a negative correlation between the two variables and the other following independent normal distributions, with different proportions (patterns N2 and N3).

### Filtering tumors by tumor purity

To identify the narrowest purity range containing half of the samples, we employed a variable-sized sliding window across purity values ranging from 0 to 1, with a step size of 0.01. Each window was required to overlap with at least half of the samples, and we selected the smallest window size as the narrowest purity range. If multiple windows exhibited the same smallest size, those with higher purity values were selected.

### Survival analysis

In this study, we conducted survival analysis by comparing two patient groups based on methylation features. We plotted Kaplan–Meier survival curves and utilized the log-rank test to assess significance. To differentiate between two patient types (hyper versus control; hypo versus control) based on the number of hypermethylated or hypomethylated probes, we employed a one-dimensional expectation–maximization algorithm. We combined two independent EM operations based on hypermethylation and hypomethylation into four patient categories: hyper, hypo, intermediate, and control. To investigate the relationship between epi-mutation burden and clinical outcomes, we selected 70% and 30% low-epi-mutation-burden samples (N_hyper_ + N_hypo_ × 4 for pattern N2 cancers, N_hyper_ + N_hypo_ × 2 for pattern N3 cancers, roughly estimated according to the distribution of hypermethylation and hypomethylation) as LowEpiMut patients for cancer types of pattern N2 and pattern N3, respectively. To exclude contamination from low-tumor-purity samples in the LowEpiMut set for pattern N2, we removed samples with tumor purities below the lower threshold of the filtered purity range.

## Results

### High heterogeneity of CpG island methylation across cancer types

**Figure 1:**
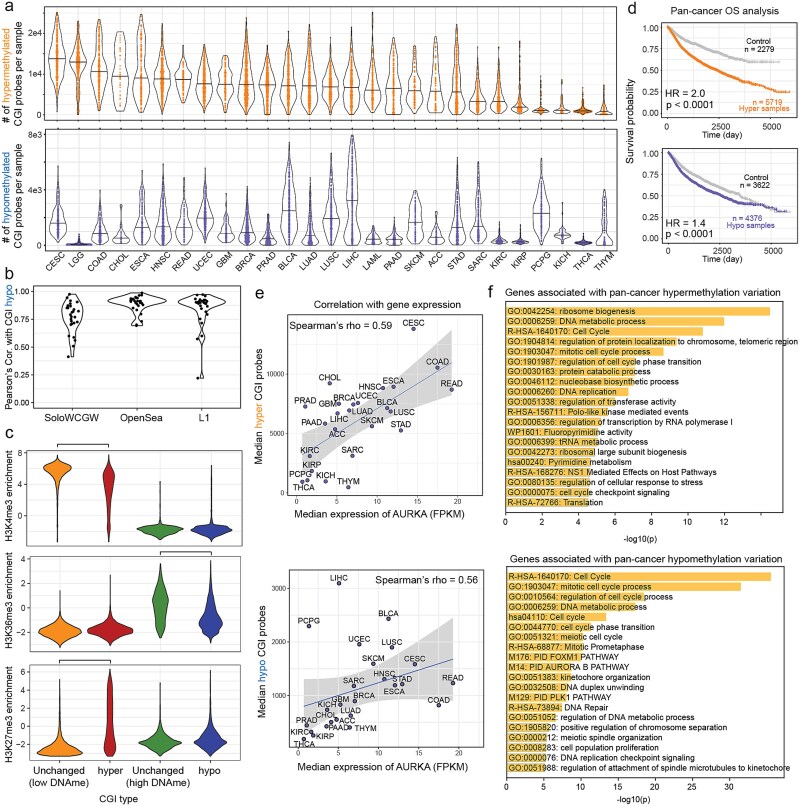
Aberrant methylation patterns of CGIs in pan-cancer. (a) Boxplots showing the numbers of hypermethylated and hypomethylated CGI probes across 27 cancer types in TCGA. Cancer types abbreviation information: https://gdc.cancer.gov/resources-tcga-users/tcga-code-tables/tcga-study-abbreviations. (b) Violin plot of Pearson’s correlations between CGI hypomethylation and widely used global hypomethylation metrics. Each point represents a cancer type. They show high correlations. (c) Histone modification enrichment for four different types of CGIs. If CGIs were consistently hypermethylated or hypomethylated in more than three cancer types, they were labeled as hyper or hypo, respectively. Hyper CGIs are enriched in H3K27me3 and depleted in H3K4me3, while hypo CGIs are depleted in H3K36me3 compared to their corresponding counterparts. (d) Kaplan–Meier curves for pan-cancer hypermethylation (top) and hypomethylation (bottom). *P*-values were calculated using the rank-sum test. (e) An example plot illustrating how to identify genes associated with hypermethylation (top) and hypomethylation (bottom) in a pan-cancer manner. The gene AURKA, involved in the cell cycle, is associated with both hypermethylation and hypomethylation (Spearman’s ρ = 0.59 and 0.56, respectively). Spearman’s correlation, rather than Pearson’s, was used to minimize the influence of extreme values from a few cancer types. (f) Top-ranked enrichment terms for the top 500 genes associated with hypermethylation and hypomethylation.

In this study, we analyzed 27 cancer types using data from the TCGA-PanCanAtlas to investigate tumor heterogeneity in CGI methylation within and across cancer types. Both hypermethylated and hypomethylated CGI probes were identified, providing an unbiased overview of CGI methylation heterogeneity across cancers (Fig. 1a; Supplementary Fig. S1). Our analysis revealed that CGI hypermethylation and hypomethylation are pervasive and highly heterogeneous. Notably, these results were not adjusted for tumor purity.

Interestingly, hypomethylation was observed in CGIs, which were previously thought to reside primarily in CpG desert regions, such as transposable elements (e.g. L1), solo-WCGWs (CpGs without flanking CpG sites within a ±35-bp window) in partially methylated domains (PMDs), and late replication regions [33, 34]. Hypomethylated CGIs were highly correlated with well-established global hypomethylation metrics, including methylation in L1, solo-WCGWs, and hypomethylated probes in open-sea regions (mean Pearson’s correlation: 0.83), suggesting a shared mechanism involving DNA methylation maintenance loss (Fig. 1b). This finding broadens our understanding of the genomic contexts associated with cancer hypomethylation.

To characterize the genomic features of aberrantly methylated CGIs, we examined histone modification enrichment. Hypomethylated CGIs were depleted for active transcription mark H3K36me3, while hypermethylated CGIs were enriched for the repressive mark H3K27me3 and depleted for promoter-associated H3K4me3 (Fig. 1c). These results suggest that most CGI methylation abnormalities in tumors are likely passive events occurring in genomic regions lacking protective epigenetic marks.

A pan-cancer survival analysis demonstrated that extreme hypermethylation and hypomethylation were associated with poor survival outcomes. CGI hypermethylation had a stronger impact on prognosis, with a hazard ratio (HR) of 2.0 compared to 1.4 for hypomethylation (Fig. 1d).

We further explored the potential biological processes associated with aberrant CGI methylation by correlating hyper- and hypomethylation levels with gene expression across cancer types. Median numbers of hyper- and hypomethylated CGI probes were calculated for each cancer type and correlated with median gene expression, excluding LGG and LAML due to their high mutation rates in methylation-related genes (Fig. 1e; Supplementary Data S1). Our analysis revealed that both hypermethylation and hypomethylation of CGIs were linked to cell cycle and DNA metabolic processes (Fig. 1f), consistent with prior findings that cell division is associated with both forms of methylation abnormalities [28–30, 32–34]. Overall, these results suggest that CGI hypermethylation and hypomethylation predominantly occur in regions lacking promoter or transcription-associated histone modifications, and are associated with cell cycle progression and poorer clinical outcomes.

### Tumor purity and the prevalence of samples with low epi-mutation burdens confound the identification of negative associations

**Figure 2:**
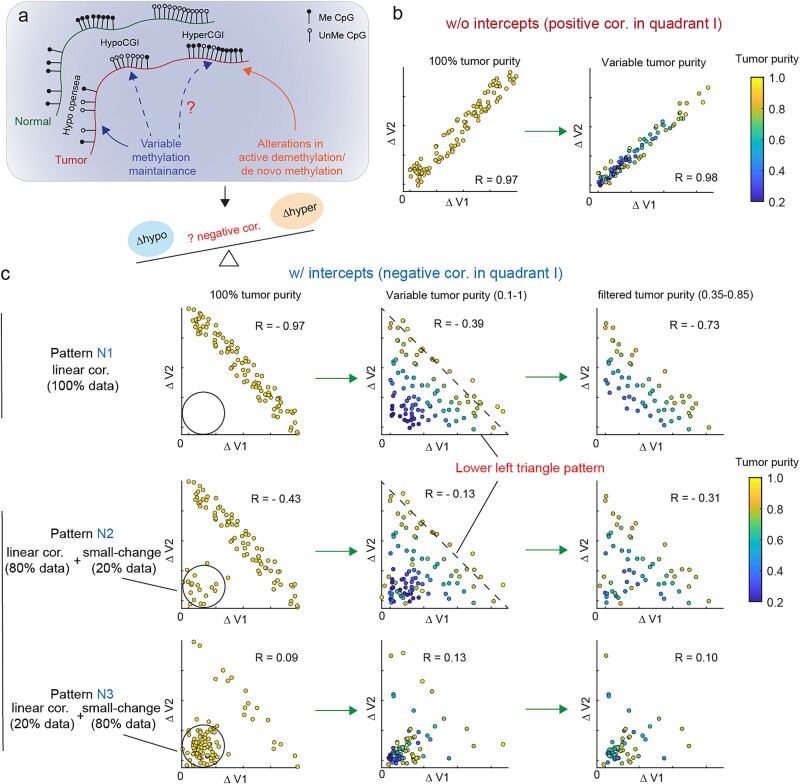
Simulation to evaluate two confounders in the identification of negative associations. (a) A schematic diagram illustrating the hypothesis that CpG island hypermethylation is affected by DNA methylation maintenance levels. This hypothesis suggests a negative correlation between alterations in hypermethylation and hypomethylation. (b) Simulation plots showing positive correlation without intercepts with axis between changes in two variables. (c) Simulation plots showing negative correlation with intercepts between changes of two variables (top) and two data mixture patterns (middle and bottom). Tumor purity and data mixture patterns largely confound the negative correlation and filtering samples to a narrow purity range alleviates this confounding.

Since CGIs without H3K36me3 protection are prone to hypomethylation due to decreased methylation maintenance, it is reasonable to hypothesize that hypermethylated CGIs are likely to be further attenuated by decreased methylation maintenance. If this is the case, a negative correlation between CGI hypermethylation and global hypomethylation, which reflects the extent of impaired methylation maintenance, would be expected (Fig. 2a). Before conducting this investigation, we need to evaluate the confounding effect of tumor purity and the mixture of low epi-mutation samples on association identification.

Tumor purity refers to the fraction of cancer cells within tumor samples, which also include stromal and immune cells. It can be estimated from both pathology slides and genomic data [41–46]. Tumor purity is a known confounder in genomic studies, particularly those focusing on tumor heterogeneity [41, 42, 47, 48]. However, previous studies on DNA methylation heterogeneity have rarely conducted comprehensive evaluations of the confounding effects of tumor purity beyond simple correlation tests. To illustrate these effects, we conducted a series of simulations (Fig. 2b, c).

First, we considered the condition of a positive correlation without intercepts with the axis (Fig. 2b). Adding the confounding effect of tumor purity did not significantly change the correlation (Pearson’s correlation, 0.98 versus 0.97). However, for negative correlations, the confounding effect of tumor purity substantially attenuated the correlation (Pearson’s correlation, −0.39 versus −0.97), resulting in a lower-left triangle pattern (Fig. 2c top). Low consistency between pathology- and *in silico*–based estimates, between DNA- and RNA-based estimates, and across different estimation methods suggests that tumor purity estimates might be qualitative and inaccurate [41, 49, 50]. Direct adjustment for tumor purity could lead to data distortion. Instead, we opted to filter samples to a narrower tumor-purity range, which improved the correlation (Pearson’s correlation, −0.73 versus −0.39), albeit at the cost of reducing the sample size and sometimes diminishing statistical power.

Additionally, we included a mixture of negatively correlated and independent small-change data points to simulate scenarios where a cancer type contained a subset of tumor samples with low epi-mutations (Fig. 2c middle and bottom). The proportion of these data points influenced the patterns imposed by tumor purity. A high fraction of negatively correlated data points in the mixture led to a weak negative correlation (Pearson’s correlation, −0.31) in the purity-filtered dataset (Fig. 2c middle). In contrast, an admixture with a low fraction of negatively correlated data points showed no or even positive correlations (Fig. 2c bottom). Overall, these simulations demonstrated that tumor purity and data mixture patterns confound the discovery of negative associations.

### Hypermethylation of CpG islands is attenuated by impaired methylation maintenance

**Figure 3:**
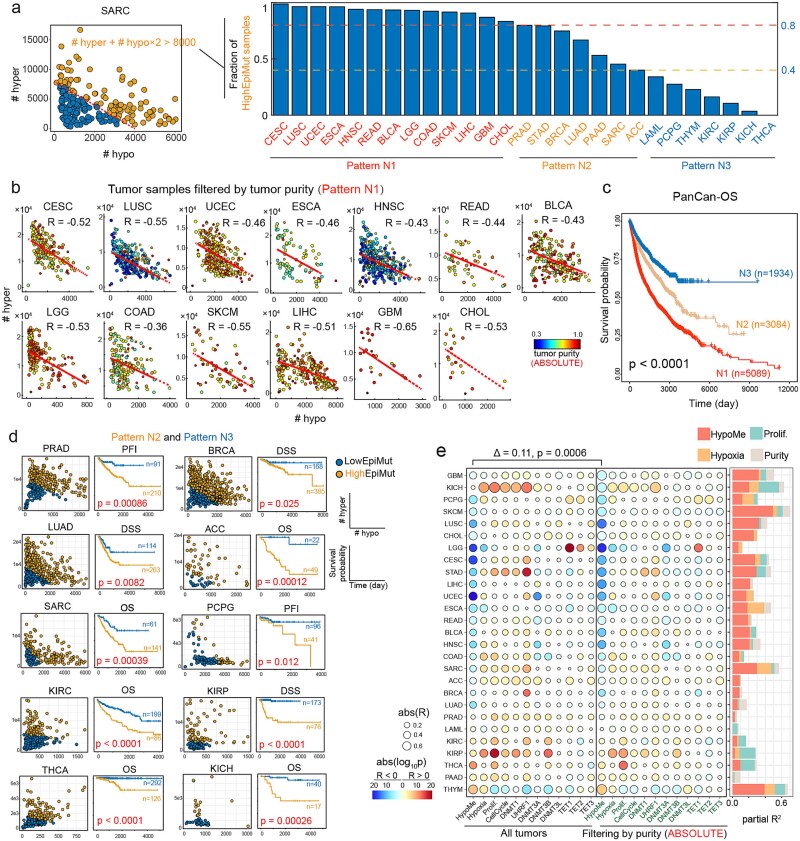
CGI hypermethylation is attenuated by impaired DNA methylation maintenance. (a) A bar plot showing the fractions of high epi-mutation (HighEpiMut) samples across 27 cancer types. Cancer types are categorized into three groups based on thresholds of 0.8 and 0.4. The left scatter plot illustrates how HighEpiMut samples are defined. (b) Scatter plots showing the numbers of hypermethylated and hypomethylated CGI probes for cancer types of pattern N1. Tumor samples (points in the plots) were filtered to satisfy that estimated purities were larger than the lowest purity threshold of the filtered sample set. Large points represent samples within the filtered purity range, while small points represent samples with purities larger than this range. (c) Kaplan–Meier survival curves for three categories of cancer types. *P*-value was calculated using a log-rank test. (d) Scatter plots showing the classification of samples into LowEpiMut and HighEpiMut types, along with associated Kaplan–Meier survival curves for cancer types of pattern N2 and pattern N3. *P*-values were calculated by log-rank tests. OS: overall survival; DSS: disease-specific survival; DFI: disease-free interval; PFI: progression-free interval. (e) A scatter plot showing the correlations between CpG island hypermethylation and heterogeneity-associated factors. Consistent negative correlations are observed between the number of hypermethylated CGI probes and hypomethylated CGI probes. Filtering samples to a concentrated purity range improved these correlations. *P*-value was calculated using a paired *t*-test. Partial *R*^2^ was calculated in the filtered tumor set only considering hypomethylation, hypoxia, proliferation, and residual tumor purity.

To exclude the confounding effects of tumor purity and the presence of low epi-mutation samples, we performed two operations. First, we evaluated the proportion of high epi-mutation samples across 27 cancer types and categorized cancers into three patterns: pattern N1 (predominantly high epi-mutation samples), and patterns N2 and N3 (containing a proportion of low epi-mutation samples) (Fig. 3a). Subsequently, we filtered tumor samples based on tumor purity in pattern N1 cancers and found that hypermethylation was significantly negatively correlated with hypomethylation, indicative of the extent of impaired methylation maintenance (Fig. 3b). Twelve out of 13 pattern N1 cancers (exception: COAD) exhibited negative Pearson’s correlations below −0.4. In contrast, only three cancers (PCPG, KICH, and STAD) in patterns N2 and N3 showed correlations less than −0.4 (Supplementary Fig. S2b, c), suggesting a confounding effect from the inclusion of samples with low epi-mutation burden.

To assess the clinical relevance of epi-mutation burdens, we conducted pan-cancer and cancer-specific survival analyses. High epi-mutation burden was linked to poor survival outcomes pan-cancer and in 10 out of 14 cancer types of patterns N2 and N3 (log-rank test, *P* < .05) (Fig. 3c, d; Supplementary Fig. S3a). Compared to CIMP classification, patient stratification by epi-mutation burden demonstrated stronger associations with clinical outcomes, notably in BRCA, LUAD, SARC, KIRP, and PRAD (Supplementary Fig. S3b, c). These results underscore the clinical relevance of epi-mutation burden derived from DNA methylation profiles.

To systematically assess the relative contributions of various factors to hypermethylation heterogeneity, we correlated hypermethylation levels with hypomethylation levels, which represent the extent of impaired methylation maintenance, and with three gene signatures (hypoxia, proliferation, and cell cycle) identified in previous studies or in our pan-cancer analysis, as well as the expression of DNA-methylation-associated genes (Fig. 3e; Supplementary Data S2). To mitigate potential bias from inaccuracies in tumor purity estimation, we incorporated five different purity estimation methods—ABSOLUTE, InfiniumPurify, MethylResolver, ESTIMATE, and CPE—which leverage distinct data types (copy number, gene expression, DNA methylation, or combinations thereof) and underlying assumptions (Supplementary Fig. S4a). After filtering for tumor purity, we observed a strengthened antagonistic relationship between hypermethylation and hypomethylation across all five estimation methods (paired *t*-test *P*-values = .0006, 8.7 × 10^−7^, .002, .06, and .01, respectively) (Fig. 3e; Supplementary Figs. S4b–d andS5). These results indicate that tumor purity is a critical confounding factor in detecting negative associations, and that the observed antagonistic relationship between hypermethylation and hypomethylation is unlikely to be an artifact of purity estimation inaccuracies. Partial *R*^2^ analysis revealed that hypomethylation was the primary contributor to CGI hypermethylation. Notably, both DNMT1 and UHRF1, key regulators of DNA methylation maintenance, were positively correlated with CGI hypermethylation, further supporting the role of DNA methylation maintenance mechanisms in shaping CGI hypermethylation patterns (Fig. 3e). We also observed that other factors emerged as the strongest independent contributors to hypermethylation variability in several specific cancers—including proliferation signature in KICH, THCA, and KIRC; *IDH1* and *DNMT3A* somatic mutations in LGG and LAML, respectively; and hypoxia signature in KIRP (Supplementary Fig. S6). These findings underscore the heterogeneous nature of methylation regulatory mechanisms across different cancer types. In conclusion, our analysis suggests that impaired DNA methylation maintenance is a major contributor to CGI hypermethylation heterogeneity across a wide range of cancer types.

### Validation of CpG island hypermethylation heterogeneity contributors using large cancer cell line data

**Figure 4:**
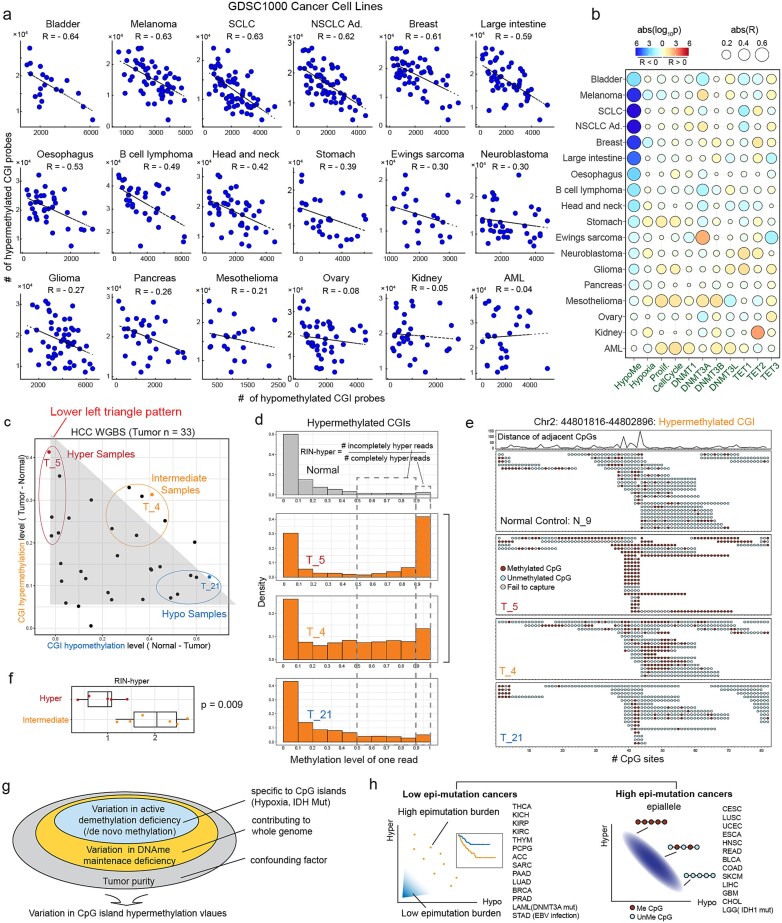
Validation of antagonistic relationship between hypermethylation and hypomethylation. (a) Scatter plots showing the numbers of hyper- and hypomethylated CGI probes in cancer cell lines. Half of the cancer types exhibit negative correlations of less than −0.4, suggesting consistent widespread correlations in cancer cell lines. SCLC: small cell lung cancer; NSCLC ad.: non-small cell lung cancer adenocarcinoma; AML: acute myeloid leukemia. (b) A scatter plot showing the correlations between CpG island hypermethylation and potential associated factors. (c) A scatter plot showing hypermethylation and hypomethylation in a WGBS cohort of hepatocellular carcinoma. Three types of tumor samples were selected to represent different methylation patterns. (d) Histograms showing methylation levels of each sequencing read located in hypermethylated CGIs for normal samples and three representative samples. A metric, RIN-hyper (ratio of incompletely methylated reads to completely methylated reads), was proposed to represent the effect imposed by DNA methylation maintenance loss. (e) Lollipop plots showing examples of a hypermethylated CGI at single-read and single-CpG-site resolution. Intermediately methylated reads are more frequent in tumor sample t_4. (f) Boxplot of RIN-hyper in hyper and intermediately methylated samples. *P*-value was calculated using two-sided *t*-test. (g) A schematic plot illustrating the sources of variation in CpG island hypermethylation in clinical tumors. (h) Two types of cancers classified by the heterogeneity patterns of CGI methylation: low epi-mutation cancers and high epi-mutation cancers.

To further validate the newly identified contributor to CpG island hypermethylation heterogeneity in a tumor purity–independent context, we analyzed data from 723 cancer cell lines in GDSC1000, which are considered highly pure in terms of cancer cell content. We examined the relationship between hypermethylation and hypomethylation, observing Pearson’s correlations of less than −0.40 in half of the cancer types (9/18) (Fig. 4a, b; Supplementary Data S3). The cancer types exhibiting weak or no correlations were consistent with those observed in the TCGA-PanCanAtlas dataset, including pancreas, kidney cancer, and acute myeloid leukemia. These findings provide independent validation of impaired DNA methylation maintenance as a novel contributor to CGI hypermethylation heterogeneity.

### Epi-allele patterns in two independent WGBS cohorts

WGBS data facilitate epi-allele level analysis, enabling us to examine the impact of impaired DNA methylation maintenance on the epi-allele patterns of hypermethylated CGIs. To validate our findings at the epi-allele level, we utilized two cohorts of WGBS data from esophageal squamous cell carcinoma and hepatocellular carcinoma (HCC). The distribution patterns of hypermethylation and hypomethylation in both cohorts closely aligned with the lower-left triangle pattern observed in the simulations (Figs. 2c and4c; Supplementary Fig. S7a), further reinforcing the relationship between CGI hypermethylation and DNA methylation maintenance levels.

Samples T_5, T_4, and T_21 from the HCC cohort represented hypermethylated, intermediately methylated, and hypomethylated states, respectively (Fig. 4c). To characterize epi-allele pattern differences across these sample types, we calculated the distribution of methylation levels for single sequencing reads mapped to hypermethylated CGIs and defined a metric, RIN-hyper, representing the ratio of incompletely hypermethylated reads (0.5 < Me <0.9) to fully hypermethylated reads (Me > 0.9) (Fig. 4d). T_4, the intermediately methylated sample, exhibited high RIN-hyper values, suggesting that DNA methylation maintenance loss might transition completely methylated epi-alleles to incompletely methylated ones (Fig. 4d, e). Statistical comparison across additional samples further supported this observation (Fig. 4f). These findings were corroborated by the esophageal squamous cell carcinoma cohort (Supplementary Fig. S7b–d).

In summary, data from three distinct resources reinforce the role of impaired methylation maintenance as a significant contributor to CGI hypermethylation heterogeneity (Fig. 4g). Moreover, based on the level of epi-mutation burden, cancers can be categorized into two types: low and high epi-mutation cancers (Fig. 4h). The former has been linked to clinical outcomes, and we are investigating the implications of the antagonistic relationship between hypermethylation and hypomethylation for cancer biomarker development.

### The implications on cancer biomarker development

**Figure 5:**
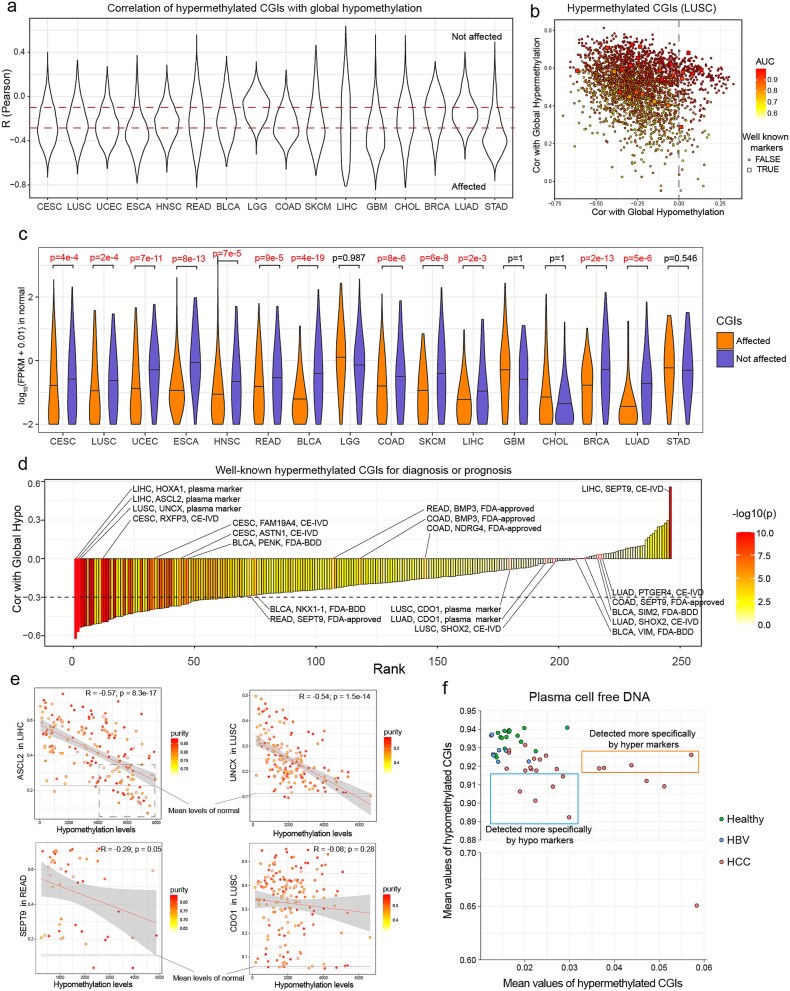
The implications for cancer biomarker development. (a) Violin plots of Pearson’s correlations between methylation of hypermethylated CGIs and global hypomethylation in 16 cancer types, which excluding cancer types with a high fraction of low epi-mutation samples. A large fraction of hypermethylated CGIs showed negative correlations. We chose two subsets of CGIs: affected and not affected by impaired methylation maintenance. (b) Scatter plot of the correlations between methylation of hypermethylated CGIs and global hypomethylation (*x*-axis) and hypermethylation (*y*-axis) in LUSC, along with the area under curve (AUC) in distinguishing tumor tissues and normal tissues. (c) The distribution of gene expression in normal tissues. CGIs not affected by impaired DNA methylation maintenance are enriched for higher expression genes. (d) The Pearson’s correlation scores between 246 well-known cancer biomarkers and global hypomethylation. In total, 65 out 246 biomarkers are significantly negatively associated (*R* < −0.3, *P* < .05) with global hypomethylation representing the level of methylation maintenance loss. (e) Scatter plots for methylation levels of four biomarkers and global hypomethylation. (f) Scatter plots for a plasma cell–free DNA dataset of HCC. Hypermethylated and hypomethylated CGIs showed their detection specificity.

Hypermethylated CGIs are widely used as biomarkers for cancer diagnosis and prognosis. To assess the impact of decreased methylation maintenance on hypermethylated CGIs, we correlated all hypermethylated CGIs with global hypomethylation level and found that a significant proportion of CGIs were affected by the antagonistic relationship between hypermethylation and hypomethylation (Fig. 5a, b). We then selected two subsets of hypermethylated CGIs: not affected CGIs (*R* > −0.1) and affected CGIs (*R* < −0.3). We found that hypermethylated CGIs unaffected by impaired methylation maintenance exhibited significantly higher expression levels in normal tissues across 12 out of 16 cancer types (one-sided *t*-test) (Fig. 5c), suggesting hypermethylation of these CGIs are likely involved in active repression during carcinogenesis rather than passive encroachment. For instance, we identified the CGI on the *CDO1* promoter, which is hypermethylated and unaffected by methylation maintenance loss in eight cancer types (CESC, LUSC, UCEC, READ, BLCA, BRCA, LUAD, STAD), consistent with *CDO1* being a known tumor suppressor.

Furthermore, we conducted two analyses to demonstrate the implications of the global antagonistic relationship between hypermethylation and hypomethylation. First, we compiled 246 hypermethylated biomarkers commonly used or tested in cancer diagnosis (several for prognosis) across 10 cancer types from published reviews [17, 18, 51–59]. In total, 65 biomarkers showed significant associations with global hypomethylation (Pearson’s correlation < −0.3, *P* < .05) (Fig. 5d; Supplementary Data S4), suggesting that these biomarkers may have limitations in detecting patients with severe methylation maintenance loss, such as *ASCL2* in LIHC, UNXC in LUSC, and *SEPT9* in READ (FDA approved, marginally significant) (Fig. 5e). However, numerous biomarkers, such as *CDO1* and *SHOX2* in LUSC and LUAD, and *SEPT9* in COAD and LIHC, were unaffected by impaired methylation maintenance (Fig. 5d, e). Second, we validated the cancer detection specificity of hypomethylation and hypermethylation biomarkers under a more stringent and clinically relevant condition—plasma cell–free DNA (Fig. 5f; Supplementary Fig. S8a, b). Together, these results underscore the importance of incorporating both hypermethylation and hypomethylation biomarkers in cancer detection.

## Discussion

This study systematically analyzed the relationship between CGI hypermethylation and impaired DNA methylation maintenance across multiple cancers, highlighting its implications for cancer biomarker design. While previous studies have alluded to this relationship—such as the low-throughput examination of the relationship between L1 and eight CGIs in colorectal cancer [60], cancer cell line knock-down experiment [21], and plots shown in pan-cancer studies [3, 10], linkage in their relative genomic positions [61, 62]—the small number of samples or the confounding effect of tumor purity and low epi-mutation samples have hindered a clear understanding of the relationship.

Although tumor purity can be estimated from both pathology and molecular data, these methods exhibit low consistency. For example, purity estimates from ABSOLUTE and InfiniumPurify show notable discrepancies, particularly in PAAD (Supplementary Fig. S4b, c). These inconsistencies can be partly attributed to the inherent limitations of the estimation methods, which are based on different assumptions. For instance, ABSOLUTE estimates purity using copy number information from SNP data, but “quiet-genome” tumors with low SNP numbers may not be accurately assessed [41]. Other methods, such as InfiniumPurify, PAMES, and MEpurity, rely on the assumption that cancer cells in all tumors exhibit constant DNA methylation alterations in selected markers, a premise that may not align with the true heterogeneity of samples, leading to biased estimates [45, 63, 64]. Overall, using a subset of samples with a narrower tumor-purity range to mitigate the confounding effects of tumor purities might be a suitable approach in cancer studies.

Previously, CpG-depleted regions were considered to be the primary targets of hypomethylation due to DNA methylation maintenance deficiency in cancer cells. Here, we identified widespread CGIs hypomethylated across cancers, extending the scope of hypomethylation from low-CpG-density regions to high-CpG-density regions. This suggested that low CpG density, or long genomic distances between adjacent CpGs, may not be the primary factors that are prone to hypomethylation. Instead, loss of protection from other epigenetic marks, such as histone modification H3K36me3, or high gene expression levels, may predispose these CGIs to passive methylation loss. Interestingly, our findings suggest that most hypermethylated and hypomethylated CGIs likely arise from passive encroachment rather than active catalysis. The antagonistic relationship between hypermethylation and hypomethylation could, however, provide valuable insights into identifying methylation cancer driver genes.

In conclusion, our study underscores the critical role of variable DNA methylation maintenance in driving CGI hypermethylation heterogeneity across various cancers. Our findings emphasize the importance of considering both hypermethylation and hypomethylation patterns, while also accounting for tumor purity and low epi-mutation samples, to better understand cancer epigenetics and develop more effective cancer biomarkers.

**Key Points**

- Tumor purity and the prevalence of low epi-mutation samples obscure the identification of negative regulators of CpG island hypermethylation, complicating the dissection of sources of heterogeneity.
- Impaired DNA methylation maintenance, reflected by global hypomethylation levels, is identified as the largest contributor to CpG island hypermethylation variability across tumors.
- A total of 65 out of 246 widely used hypermethylation biomarkers are significantly influenced by impaired methylation maintenance, affecting their reliability across 10 cancer types.
- Incorporating both hypermethylation and hypomethylation biomarkers in cancer detection improves robustness, as validated using plasma cell–free DNA datasets from cancer patients, showcasing potential for advancing diagnostic applications.

## Supplementary Material

supplementary_information_bbaf252

Supplementary_Data_1_bbaf252

Supplementary_Data_2_bbaf252

Supplementary_Data_3_bbaf252

Supplementary_Data_4_bbaf252

## Data Availability

DNA methylation, somatic mutations, and gene expression data of TCGA were downloaded from GDC data portal (https://portal.gdc.cancer.gov/) [66]. Clinical survival outcome data of TCGA were downloaded from TCGA-PanCanAtlas (https://gdc.cancer.gov/about-data/publications/pancanatlas) [67]. Tumor purity estimates for TCGA samples were obtained from https://gdc.cancer.gov/about-data/publications/pancanatlas (ABSOLUTE), https://doi.org/10.1038/ncomms3612 (ESTIMATE), https://zenodo.org/records/253193 (InfiniumPurify), https://doi.org/10.1038/s42003-020-01146-2 (MethylResolver), and https://doi.org/10.1038/ncomms9971 (CPE) [42, 44–46, 68]. External normal samples with Infinium HumanMethylation450 BeadChip were obtained from Gene Expression Omnibus (GEO) of NCBI (Normal adrenal tissue: GSE77871; Control brain: GSE79122; bone marrow CD34^+^ cells: GSE58477; Cord blood samples including B cells, CD4 and CD8 T cells, granulocytes, monocytes, NK cells, and nucleated RBCs: GSE68456; Nevus sample: GSE120878; Epithelial layer of resected ovaries: GSE146552) [69–74]. DNA methylation data and gene expression data for GDSC1000 cancer cell lines were downloaded from GEO (GSE68379) and https://www.cancerrxgene.org, respectively [75, 76]. Two WGBS cohorts of tumors with corresponding normal samples were downloaded from Sequence Read Archive of NCBI (esophageal squamous cell carcinoma: PRJNA523898; hepatocellular carcinoma: PRJNA762641) [77, 78]. Plasma cell–free DNA datasets were downloaded from European Genome–Phenome Archive (EGA) with accession no. EGAS00001000566 [65] and GEO with accession nos. GSE79279 [79] and GSE149438 [80]. Histone modification data (H3K4me3, H3K36me3, H3K27me3) of H1 stem cell line were downloaded from ENCODE portal (https://www.encodeproject.org/) [81].

## Code availability

All source code is accessible at https://github.com/vhang072/MeHist.
