# Supplementary material for: Simulation-guided pan-cancer analysis identifies a novel regulator of CpG island hypermethylation heterogeneity
Source: Brief Bioinform. 2025 Jun 2;26(3):bbaf252. doi: 10.1093/bib/bbaf252 (PMC12127147; doi:10.1093/bib/bbaf252)
Supplement: supplementary_information_bbaf252 [file supplementary_information_bbaf252.pdf]

Supporting Information

Simulation-Guided Pan-Cancer Analysis Identifies a Novel Regulator of CpG Island Hypermethylation Heterogeneity

Xianglin Zhang[†], Wei Zhang[†], Jinyi Zhang, Xiuhong Lyu, Haoran Pan, Tianwei Jia, Ting Wang *, Xiaowo Wang *, Haiyang Guo*

[†]These authors contributed equally

*To whom correspondence should be addressed. E-mail: haiyang.guo@email.sdu.edu.cn, xwwang@tsinghua.edu.cn, twang@wustl.edu

Supplementary Fig. 1-8

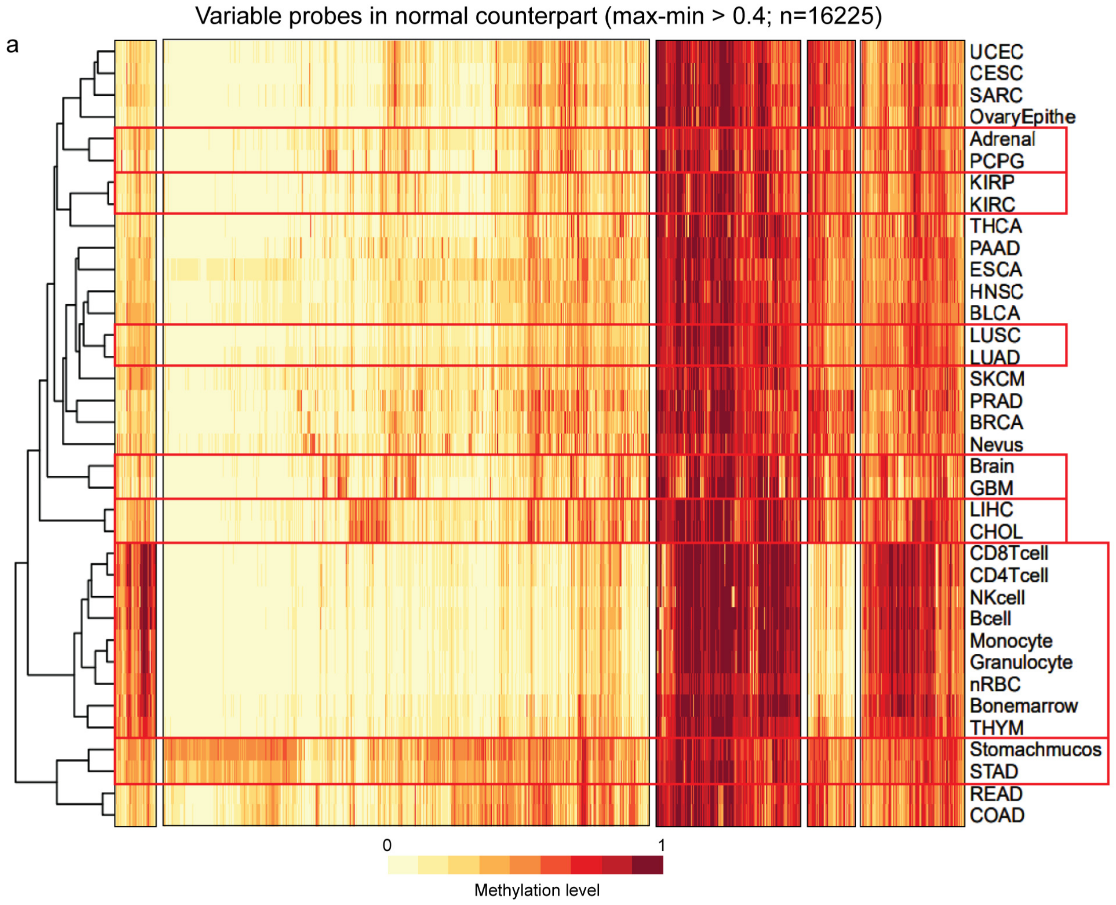

Supplementary Fig. 1 Identification of Differentially Methylated CGI Probes

(a) Clustering heatmap depicting normal counterparts from TCGA and GEO. Normal counterparts of the same type from TCGA and GEO exhibit highly consistent patterns.

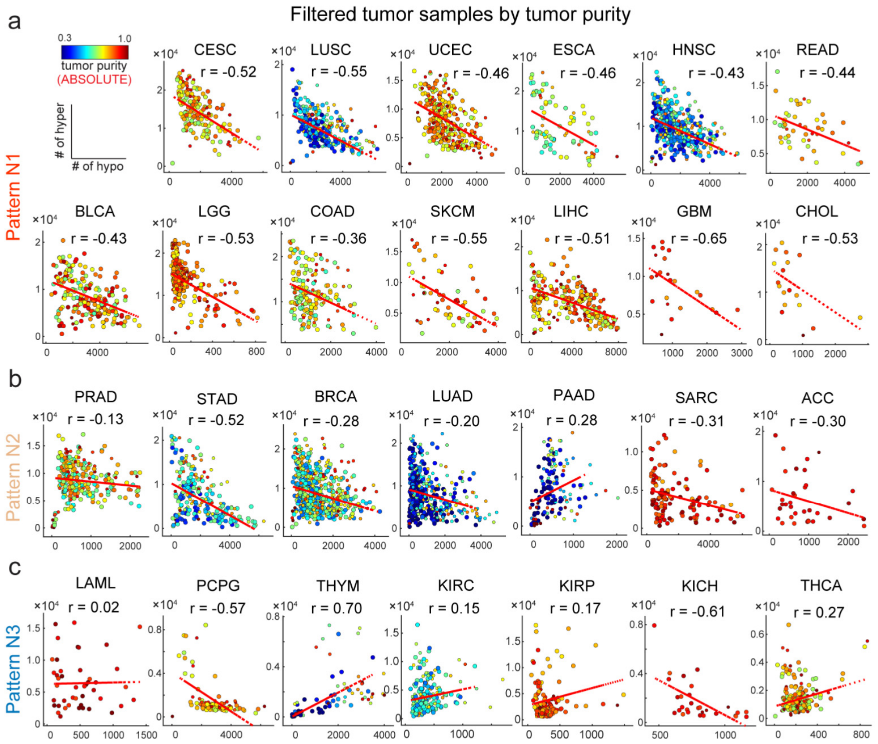

Supplementary Fig. 2 correlation between hypermethylation and hypomethylation

(a, b, c) Scatter plots showing the numbers of hypermethylated and hypomethylated CGI probes for cancer types of patterns N1(a), N2 (b), and N3 (c). Tumor samples (points in the plots) were filtered to satisfy that estimated purities were larger than the lowest purity threshold of the filtered sample set. Large points represent samples within the filtered purity range, while small points represent samples with purities larger than this range.

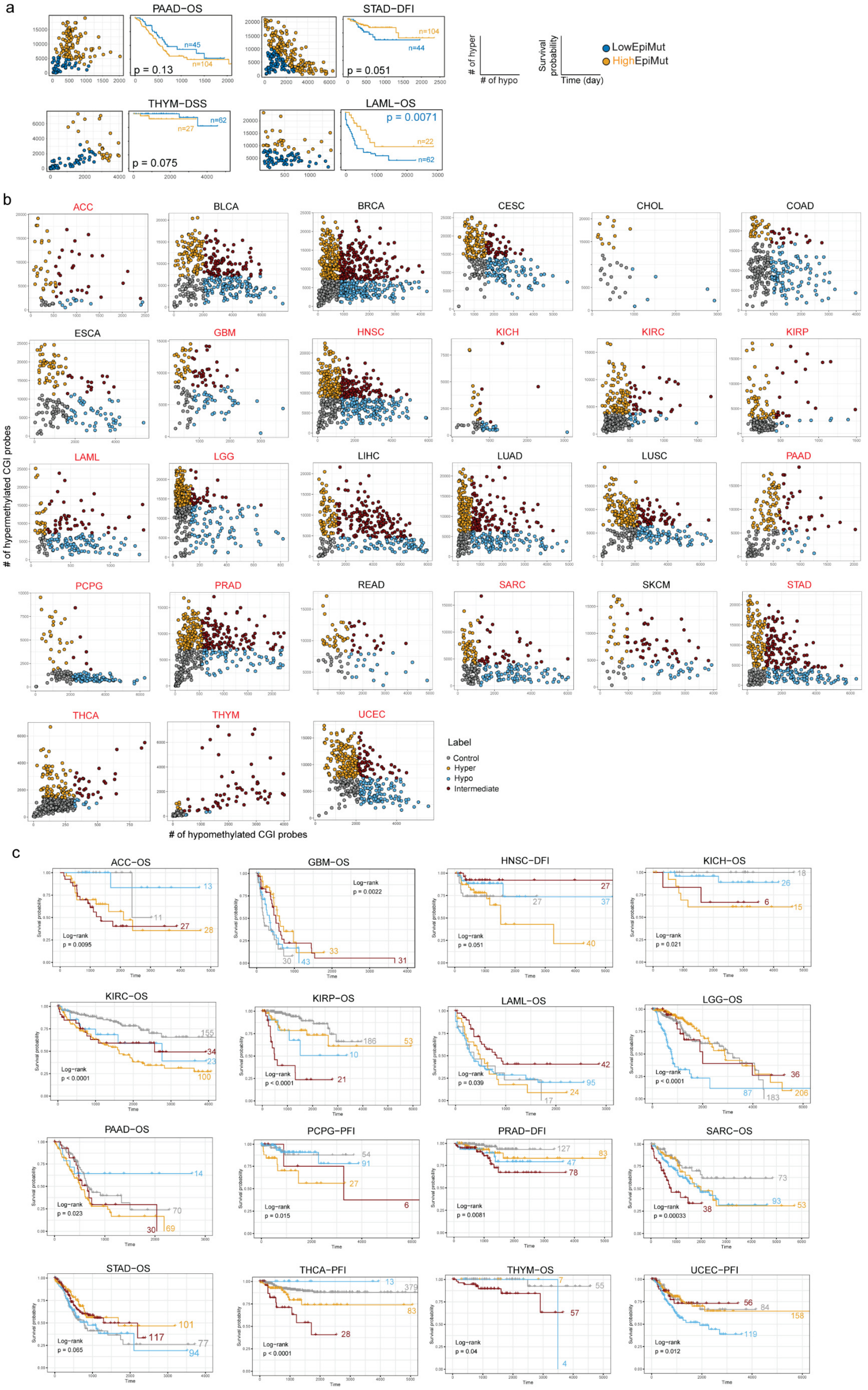
a
PAAD-OS
n=45
n=104
p = 0.13
STAD-DFI
n=104
n=44
p = 0.051
THYM-DSS
n=62
n=27
p = 0.075
LAML-OS
p = 0.0071
n=22
n=62
# of hyper
# of hypo
Survival probability
Time (day)
LowEpiMut
HighEpiMut
b
ACC
BLCA
BRCA
CESC
CHOL
COAD
ESCA
GBM
HNSC
KICH
KIRC
KIRP
LAML
LGG
LIHC
LUAD
LUSC
PAAD
PCPG
PRAD
READ
SARC
SKCM
STAD
THCA
THYM
UCEC
Label
Control
Hyper
Hypo
Intermediate
# of hypermethylated CGI probes
# of hypomethylated CGI probes
c
ACC-OS
Log-rank
p = 0.0095
GBM-OS
Log-rank
p = 0.0022
HNSC-DFI
Log-rank
p = 0.051
KICH-OS
Log-rank
p = 0.021
KIRC-OS
Log-rank
p < 0.0001
KIRP-OS
Log-rank
p < 0.0001
LAML-OS
Log-rank
p = 0.039
LGG-OS
Log-rank
p < 0.0001
PAAD-OS
Log-rank
p = 0.023
PCPG-PFI
Log-rank
p = 0.015
PRAD-DFI
Log-rank
p = 0.0081
SARC-OS
Log-rank
p = 0.00033
STAD-OS
Log-rank
p = 0.065
THCA-PFI
Log-rank
p < 0.0001
THYM-OS
Log-rank
p = 0.04
UCEC-PFI
Log-rank
p = 0.012
Survival probability
Time

Supplementary Fig. 3 Integrative survival analysis of hypermethylation and hypomethylation

(a) Scatter plots showing the classification of samples into LowEpiMut and HighEpiMut types, along with associated Kaplan-Meier survival curves for cancer types PAAD, STAD, THYM, and LAML.

(b) Scatter plots of tumors based on the numbers of hyper- and hypo-methylated CGI probes. Tumors are classified into four groups based on independent clustering (EM algorithm) of hyper- and hypo-methylation. Intermediately methylated samples refer to those classified as both hyper- and hypo-methylated in the two independent clustering analyses.

(c) Kaplan-Meier survival plots for significant cancer types. Note that extremely aberrantly methylated samples, whether hypermethylation or hypomethylation, are associated with poor clinical outcomes in certain cancer types (KIRC, KIRP, PCPG, PRAD, SARC).

a

Tumor purity estimation methods	Data based	Detail Methods
ABSOLUTE	Copy number data	
InfiniumPurify	DNA methylation	Using top ranked differentially methylated CpG sites between tumor and normal
MethylResolver	DNA methylation	Inferring leukocyte subset fractions from methylation profiles
ESTIMATE	Gene expression	
CPE	Multiple purity data (ABSOLUTE, ESTIMATE, LUMP, IHC)	

Same omic data used in this study

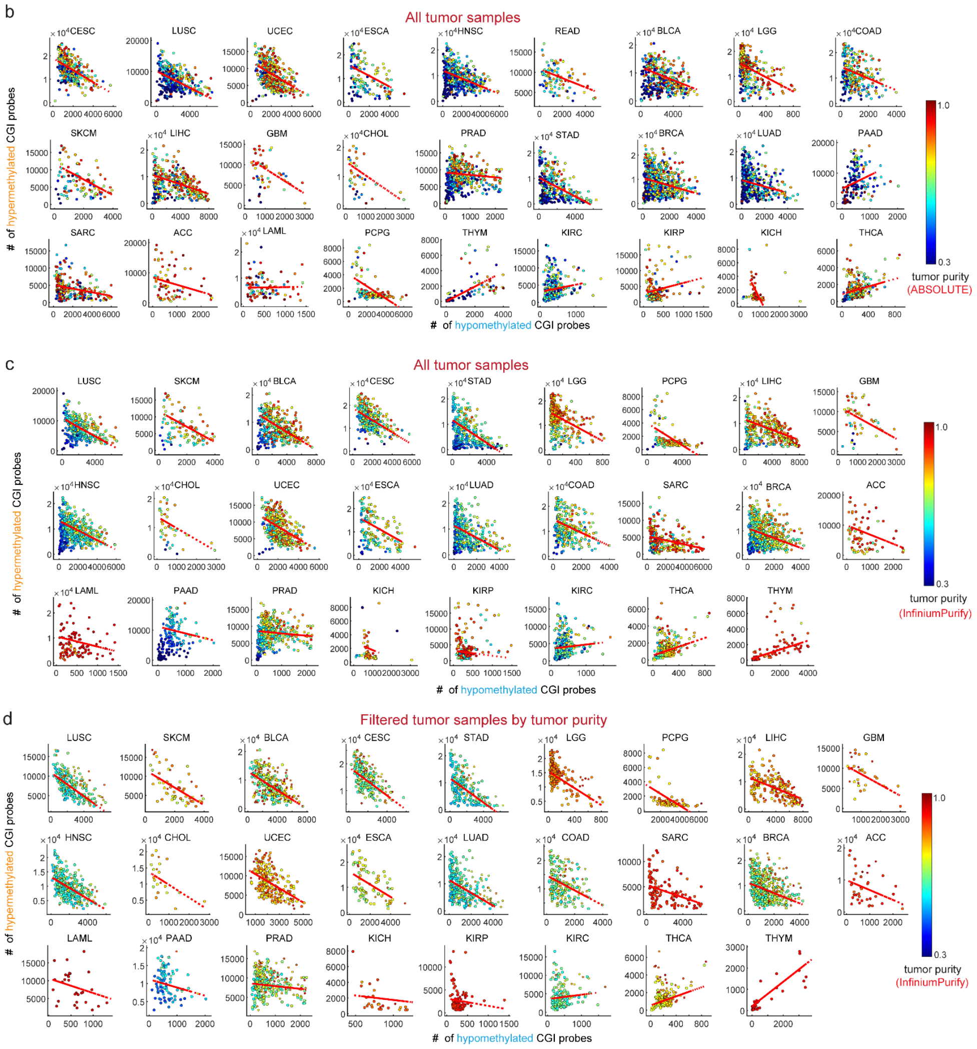

Supplementary Fig. 4 The impact of tumor purity on relationship discovery in TCGA

(a) Tumor purity estimates for TCGA samples utilized in this study. It is important to note that ABSOLUTE estimated tumor purity using SNP array data, which primarily derived from the same analyte samples (the same sample, vial, portion, and analyte) as the DNA methylation data. This avoids inconsistencies arising from the use of different materials in SNP array and DNA methylation libraries. InfiniumPurify and MethylResolver utilized the same data as our study.

(b) Scatter plots depicting the number of hypermethylated and hypomethylated CGI

probes, colored by tumor purity from ABSOLUTE. All tumors are represented.

(c) Scatter plots depicting the number of hypermethylated and hypomethylated CGI probes, colored by tumor purity from InfiniumPurify. All tumors are represented.

(d) Scatter plots depicting the number of hypermethylated and hypomethylated CGI probes, color-coded by tumor purity from InfiniumPurify. Note that points (tumor samples) shown in these plots represent samples with estimated purities exceeding the lowest threshold of filtered samples (with a more concentrated purity range in half of samples). Larger points correspond to samples with estimated purities within the filtered concentrated range, while smaller points represent samples with estimated purities exceeding that range.

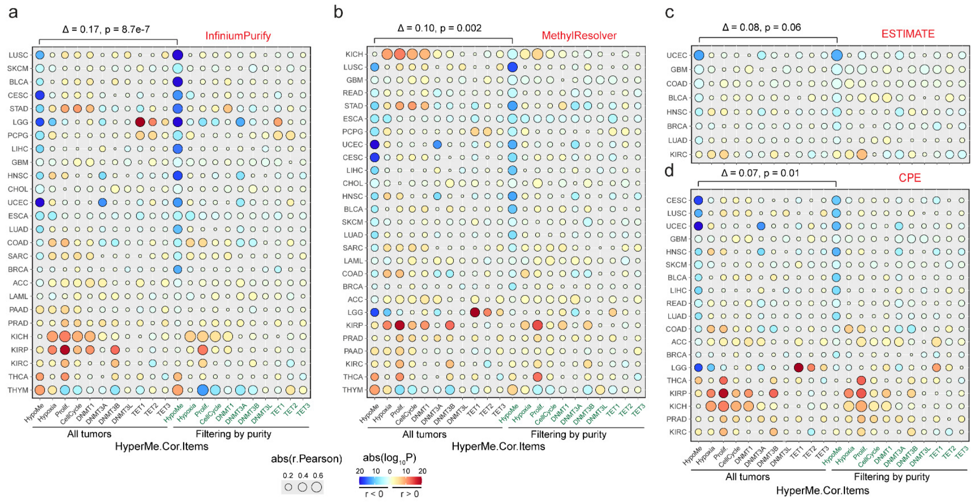

Supplementary Fig. 5 Factors contributing to CGI hypermethylation heterogeneity under different tumor purity estimates

(a-c) Scatter plots illustrating the correlations between CpG island hypermethylation and potential associated factors in a filtered sample-set with different purity estimates (InfiniumPurify, MethylResolver, ESTIMATE and CPE). Filtering samples to a concentrated purity range enhanced the correlation across all four estimates. P-values were calculated using a paired t-test.

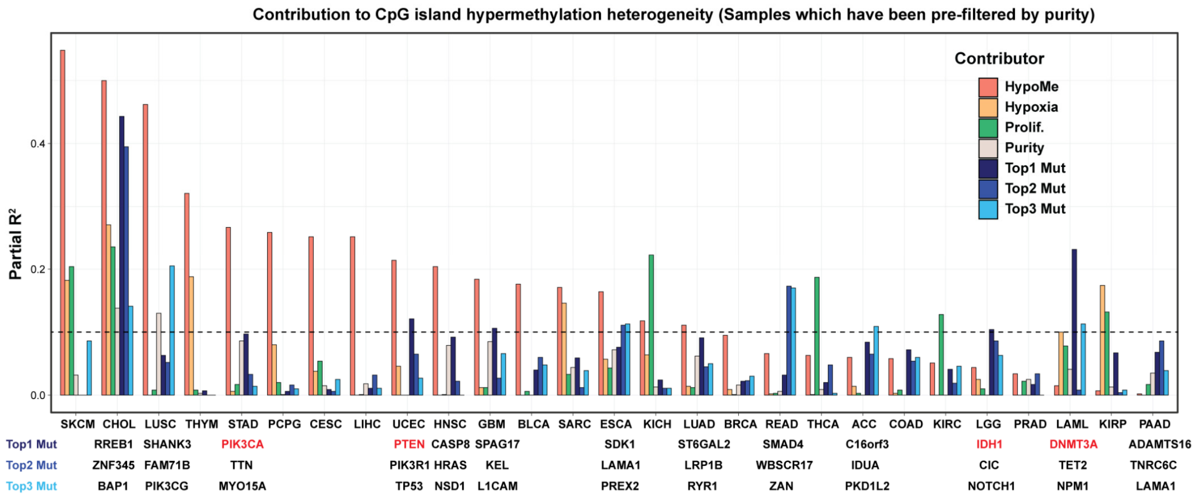

Supplementary Fig. 6 Partial R^2 of factors (hypomethylation extent, hypoxia, proliferation signature, residual factor—purity, as well as top 3 gene mutants) contributing to CGI hypermethylation heterogeneity

Top three gene mutations the most significantly associated with hypermethylation were prioritized in each cancer type using Wilcoxon tests. We then calculated the partial R^2 values (representing the percentage of independently explained variance beyond other factors) for these top mutations, as well as for hypomethylation level, hypoxia signature, proliferation signature, and tumor purity (as a residual factor). Gene names of the top 3 mutations were labeled when their partial R^2 near or larger than 0.1. Gene mutations that have been reported to be associated with hypermethylation heterogeneity were labeled in red.

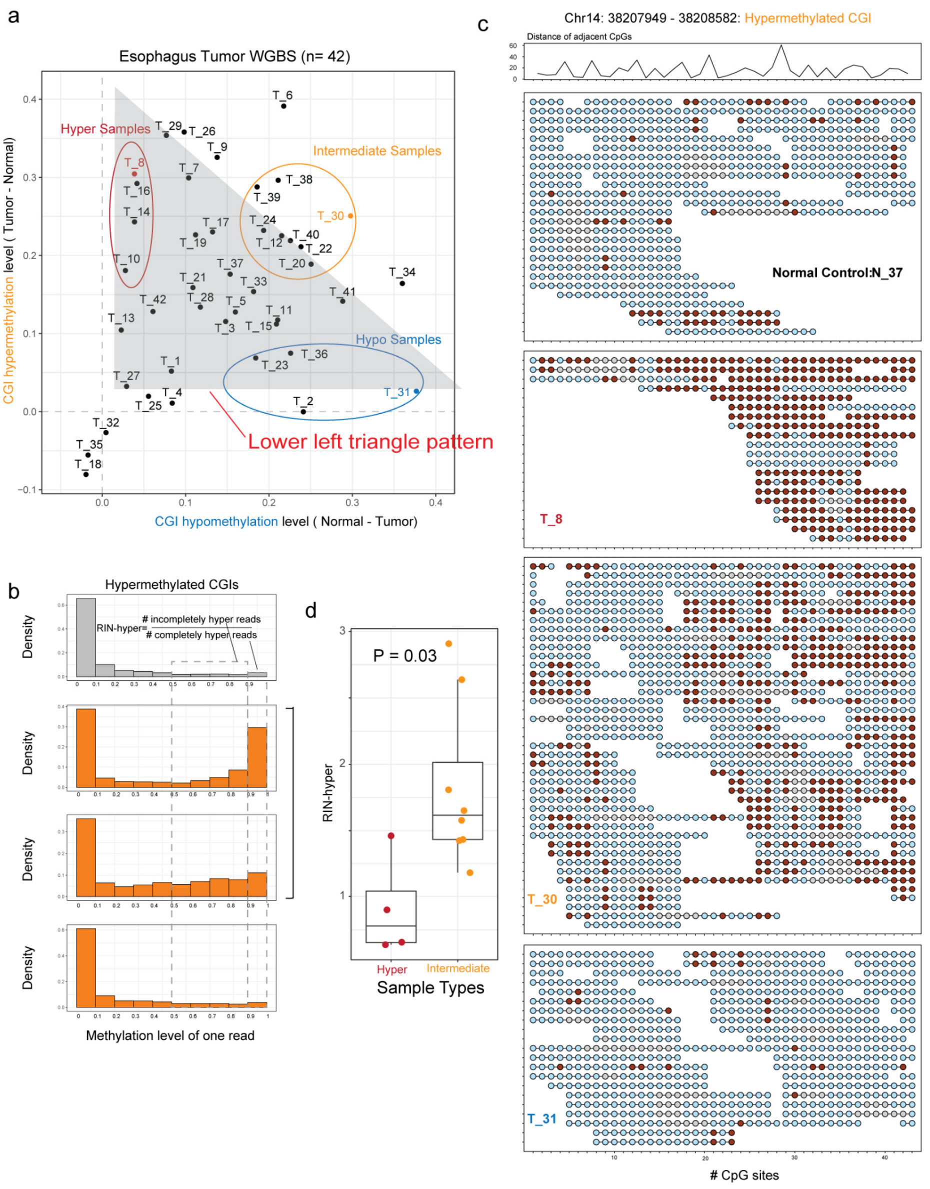

Supplementary Fig. 7 DNA methylation maintenance loss attenuates complete hypermethylation patterns of epi-alleles in a WGBS cohort of esophageal squamous cell carcinoma

(a) A scatter plot displaying hypermethylation and hypomethylation in a WGBS cohort of esophageal squamous cell carcinoma. Three types of tumor samples were selected to represent various methylation patterns.

(b) Histograms of methylation levels of each sequencing read located in hypermethylated CGIs for normal samples and three representative samples.

(c) Lollipop plots illustrating examples of a hypermethylated CGI at single-read and single-CpG-site level. Intermediately methylated reads are more prevalent in tumor sample T_30.

(d) Boxplots of RIN-hyper in hyper and intermediately methylated samples. P-values were calculated using two-sided t-tests.

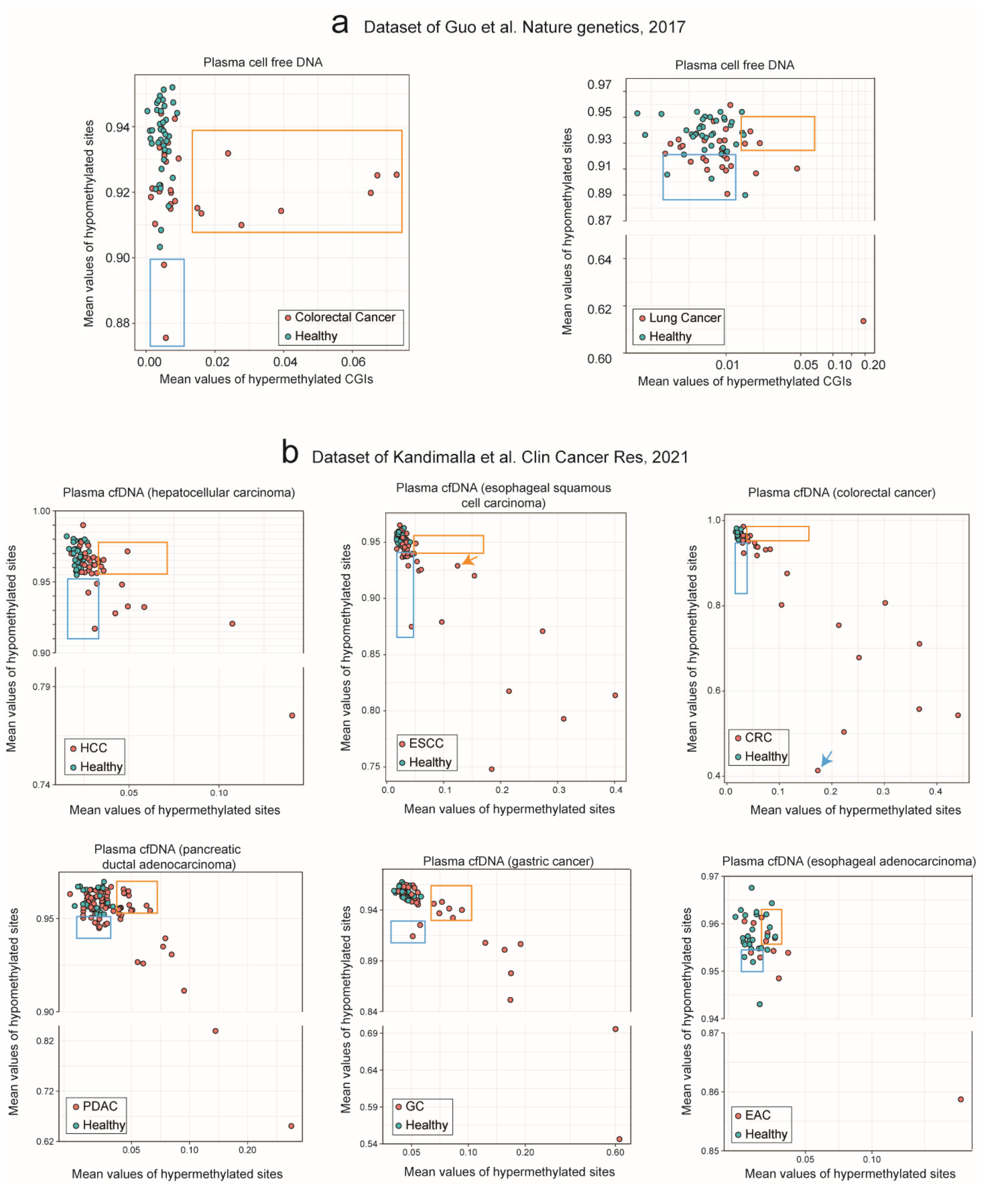

Supplementary Fig. 8 Cell-free DNA datasets showing the cancer detection specificity of hypermethylation and hypomethylation markers

(a) RRBS plasma cell-free DNA dataset of Guo et al.

(b) Personalized targeted sequencing plasma cell-free DNA dataset of Kandimalia et al.
